# Design and Fabrication of an Underwater Transducer Based on the Shear Vibration Mode and Trapezoid Transition Layer

**DOI:** 10.3390/mi13081320

**Published:** 2022-08-15

**Authors:** Yali Qiao, Shaojia Jin, Chao Zhong, Lei Qin

**Affiliations:** 1 Beijing Key Laboratory for Sensor, Beijing Information Science & Technology University, Beijing 100192, China; 2 Beijing Key Laboratory for Optoelectronic Measurement Technology, Beijing Information Science & Technology University, Beijing 100192, China; 3 Key Laboratory of Modern Measurement & Control Technology, Ministry of Education, Beijing Information Science & Technology University, Beijing 100192, China

**Keywords:** piezoelectric ceramics, shear vibration mode, transducer array, transmitting voltage response

## Abstract

In this study, a new kind of underwater transducer was developed using the $d_{15}$ shear vibration mode of piezoelectric ceramic and a trapezoid transition layer. A series of finite element simulations were conducted to investigate how the boundary conditions of piezoelectric ceramic blocks affect the shear vibration. Finite element simulation was also used to investigate how the trapezoid transition layer transfers shear vibrations into longitudinal vibrations. A prototype of the proposed transducer was fabricated from piezoelectric vibrators working in the shear mode and a trapezoid transition layer. The underwater performance of this transducer was then tested. The results demonstrated that the transmitting voltage response, working frequency range, and bandwidth reached 163 dB (62 kHz), 37 kHz–68 kHz, and 31 kHz when the radiating area of the transducer was 120 mm × 240 mm. The transmitting voltage response caused by the $d_{15}$ shear vibration mode reached 160.9 dB at 89 kHz.

## 1. Introduction

Transducers have important applications in underwater acoustic detection, ultrasound nondestructive testing, and sensing fields. The main transduction materials in these transducers are piezoelectric materials. Currently, piezoelectric materials capable of longitudinal stretching mode ($d_{33}$ mode), such as 1–3 type piezoelectric composite, have been widely used in high-frequency transducers for their characteristics of simple preparation technology, strong piezoelectricity, and large electromechanical coupling factor [1,2]. Piezoelectric materials capable of the thickness expansion mode (kt mode) or radial expansion mode ($d_{31}$ mode) have been widely used in low-frequency transducers, such as tonpilz transducers and flextensional transducers [3,4].

Other than $d_{33}$, $d_{31}$, and kt, shear vibration mode is another kind of basic vibration mode. Some specific ceramics and single crystals have relatively high shear piezoelectric constants and shear electromechanical coupling factors. For some kinds of piezoelectric ceramics, such as the PZT-5A and PZT-5H types, $d_{15}$ is significantly higher than $d_{33}$, while $k_{15}$ and $k_{33}$ are equivalent. In one study, the $k_{15}$ of xPb(In_1/2_Nb_1/2_)O_3_-yPb(Mg_1/3_Nb_2/3_)O_3_-zPbTiO_3_ (PIMNT) single crystal with [110] orientation reached 96.4%, and the $d_{15}$ mode was 5966 pC/N when platinum electrodes were used [5]. Therefore, the reasonable selection of piezoelectric materials and application of $d_{15}$ mode have the potential to improve the performance of piezoelectric transducers.

At present, there are few studies on the $d_{15}$ mode of piezoelectric materials, which is mainly applied in energy harvesting, actuators, and new structural composites. For instance, Dong et al. designed a piezoelectric energy harvester with an L-shaped base that uses the $d_{15}$ mode. Through the collaborative effects of a mass block, piezoelectric plate, and base, this piezoelectric energy harvester transforms acceleration into a shear force through the mass block upon excitation, and this shear force acts on the piezoelectric plate. This structure has relatively high output power [6]. Zheng et al. studied a PZT piezoelectric bimorph cantilever beam device based on the serial-parallel structure of the $d_{15}$ mode, which further increased the output power [7]. Gao et al. proposed a multilayer cylindrical piezoelectric actuator working in shear mode, which consists of piezoelectric ceramic rings polarized in parallel along the axial direction with alternating positive and negative poles. Compared with the actuators working in other modes, the maximum driving force is increased by seven times [8]. Miao et al. proposed a face-shear $d_{24}$ PZT wafer to excite and receive a pure SH0 wave in plate structures. This wafer can also serve as a selective SH wave sensor, which can exclude lamb waves from 120 to 230 kHz [9]. Kranz et al. proposed a new piezoelectric $d_{15}$ shearing macroscopic fiber composite (MFC) with longitudinal polarization. Under electric field excitation conditions, the shearing MFC presented better electromechanical coupling efficiency [10].

To sum up, the shear mode of piezoelectric ceramics has been applied in various fields, due to the unique advantages of tangential deformation, higher piezoelectricity, and electromechanical coupling factors. It can be seen that $d_{15}$ shear vibration mode has great research value and broad application prospects. However, since shear vibrations cannot radiate in water, the shear vibration mode has not been applied in underwater transducers.

With respect to the disadvantage of the shear vibration mode’s inapplicability to acoustic emission in water, a trapezoid transition layer has been proposed to transform shear vibrations of piezoelectric materials into thickness vibrations of the transition layer, thus realizing acoustic emission [11]. Based on the above research results, in order to explore the application prospect of shear vibration of piezoelectric materials in the field of underwater acoustic transducers, this paper proposes to use the shear vibration mode of piezoelectric ceramics to prepare underwater acoustic transducers. By introducing the trapezoidal transition layer structure in the maximum strain region of the piezoelectric ceramic shear vibration, the shear vibration of the piezoelectric ceramic is transformed into the surface vibration of the transition layer, so as to realize the effect of sound wave propagation to water. This study optimized the structure of an underwater transducer, based on the shear mode, by replacing the back transition layer with a positioning bar. Furthermore, a series of simulation models on a single element, four bonding elements, and the whole structure were conducted to identify how these boundary conditions affect the shear vibration. Finally, the ability of the trapezoid transition layer to transform shear vibrations into longitudinal vibrations was evaluated. This study provides a new design idea for piezoelectric transducers based on shear vibration mode.

## 2. Structure of Oscillator and Transducer

### 2.1. Structure of Oscillator

**Figure 1:**
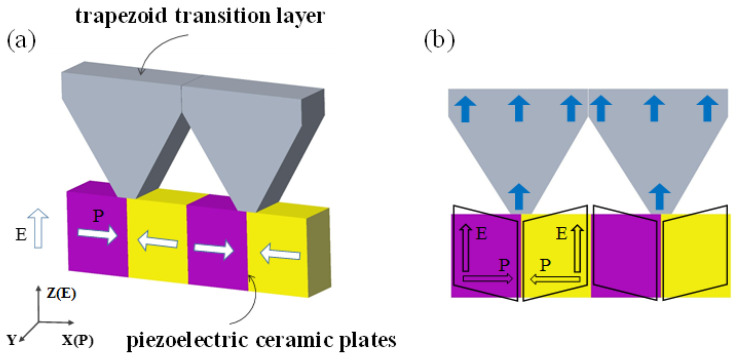
Piezoelectric oscillator based on shear vibration mode. (**a**) Structure diagram; (**b**) vibration form of the oscillator.

The proposed piezoelectric oscillator structure based on shear vibration mode is shown in Figure 1a. This vibrator is composed of piezoelectric ceramic plates and a trapezoid transition layer. Specifically, the piezoelectric ceramic is polarized along the X-axis (length direction), and the polarization directions of two adjacent piezoelectric ceramic plates are opposite. Piezoelectric ceramics were bonded along the length direction, and the electrodes were prepared along the upper and lower surfaces (width directions) along the Z-axis. A trapezoid transition layer was pasted at the ceramic joints directly. With consideration of impedance matching and machining characteristics, duralumin was selected to be the transition layer material.

The vibration mode of the piezoelectric oscillator is shown in Figure 1b, where P is the polarization direction, and E is the direction of the electric field. An electric field was applied perpendicular to the polarization direction of piezoelectric ceramics, which would excite the $d_{15}$ mode of the piezoelectric ceramics and produce shear vibration. The maximum displacement of this vibration was mainly concentrated at the ceramic joints. This vibration was then transmitted to the surface of the transition layer through the upper trapezoid transition layer, thus realizing the transformation of shear vibrations of piezoelectric ceramics into the longitudinal mode.

### 2.2. Structure of Transducer

**Figure 2:**
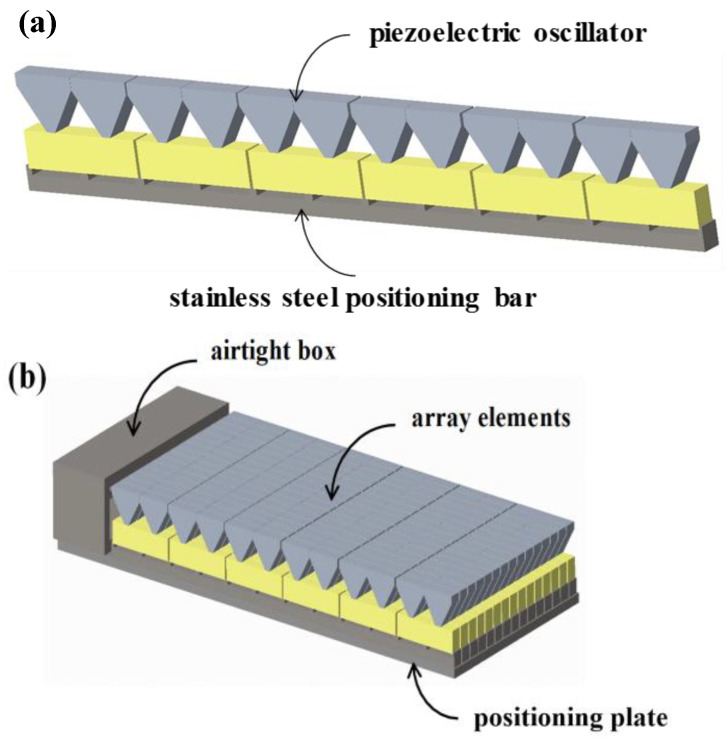
Structure of array element and underwater transducer based on shear mode. (**a**) One element of the underwater transducer; (**b**) 6 × 14 rectangular transducer array.

In this study, piezoelectric oscillators, of the type shown in Figure 1, were used as basic vibrators. As shown in Figure 2a, by arranging six oscillators into a stainless-steel positioning bar, one element of the underwater transducer was formed. In addition, a transducer was designed according to certain forms of arrangement and combination through several array elements, thus increasing the radiation area and improving various performance metrics, such as transmitting voltage response and the impedance of a transducer.

The internal structure of the designed underwater transducer is shown in Figure 2b. First, 14 array elements were arranged reasonably at equal distances and then fixed on the positioning plate. They formed a 6 × 14 rectangular transducer array. Subsequently, all leads of the array were welded onto the output cable through an airtight box. The airtight box was equipped with a waterproof rubber gasket. Next, this array was put into a filling mold. Finally, the array was sealed with polyurethane (PU).

## 3. Finite Element Simulation Analysis

A series of finite element simulations were carried out using finite element analysis software ANSYS (ANSYS, Inc., Canonsburg, PA, USA). FEA (finite element analysis) is an efficient numerical calculation method based on variational principle and subdivision interpolation, combined with mathematical and physical approximation to calculate and simulate real physical systems [12]. In order to study how the boundary conditions of the piezoelectric ceramic blocks affected the shear vibration, the finite element simulations included a single element, four elements, and four elements with a trapezoid transition layer. Since the proposed transducer is formed via the repeated arrangement and combination of multiple piezoelectric oscillators, it only has to apply a symmetric boundary condition to a single piezoelectric oscillator. As discussed in previous work, the highest electromechanical coupling factor for PZT-5A piezoelectric ceramic working at the $d_{15}$ mode was achieved when the size ratio along the electric field direction and polarization direction approached 1:1.2 [13]. In this study, a PZT-5A piezoelectric ceramic plate, which was 10 mm long (polarization direction), 12 mm wide (electric field direction), and 7 mm thick, was chosen as the active element. To consider both the operation time and accuracy, the model was meshed according to 1/10 of the wavelength. The frequency range was chosen as 30–150 kHz, and the constant damping ratio was set to 0.02. The material parameters of the piezoelectric oscillator are shown in Table 1. The size parameters of the piezoelectric oscillator are shown in Table 2.

### 3.1. Finite Element Analysis of a Single Element

**Figure 3:**
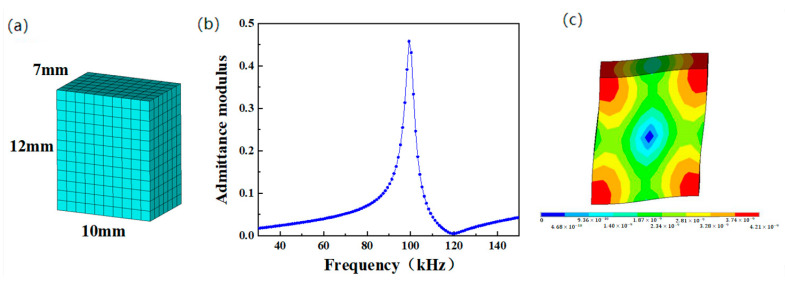
Finite element simulation results of single ceramic element. (**a**) Mesh model; (**b**) admittance modulus curve; (**c**) vibration mode.

The meshed model of a single element is shown in Figure 3a. The harmonic response was analyzed, and the quantity of electric charge on the ceramic electrode surface was obtained from the post-processing module of ANSYS. On this basis, the admittance modulus curve of the single element was calculated and is shown in Figure 3b. It was calculated from the conductance curve that the resonance frequency, anti-resonance frequency, and electromechanical coupling factor were 99.34 kHz, 119.21 kHz, and 0.59, respectively. It was a pure harmonic peak with no interferences from other modes. The vibration mode of the piezoelectric ceramic is shown in Figure 3c. The different colors in Figure 3c reflect the quantity of vibration displacement, where the quantity of vibration displacement increases from blue to red. Since the electric field was perpendicular to the polarization direction, the shear vibration mode of the ceramics was excited. The maximum strain of this vibration was mainly concentrated at the four vertexes of the ceramics, whereas the nodal point was in the center of the ceramics. These are the characteristics of shear vibration.

### 3.2. Finite Element Analysis of Four Elements

**Figure 4:**
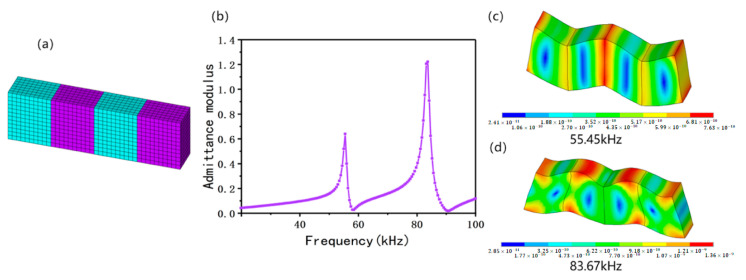
Finite element simulation results on bonding of four elements. (**a**) Mesh model; (**b**) admittance modulus curve; (**c**) vibration mode @55.45kHz; (**d**) vibration mode @83.67kHz.

Moreover, a finite element model with bonding of four elements was constructed. The meshed model is shown in Figure 4a. It also can be seen from the admittance modulus curve that two harmonic peaks at 55.45 and 83.67 kHz were produced in the frequency range of 30–100 kHz after four elements were bonded (Figure 4b). The anti-resonance frequencies and electromechanical coupling factors for both two harmonic peaks were 58.42 kHz, 0.35 and 90.60 kHz, 0.42, respectively. As we can see, after bonding four elements together, the electromechanical coupling factor dropped from 0.59 to 0.42, and the resonance frequency dropped from 99.34 to 83.67 kHz. The vibration modes corresponding to these two harmonic peaks were observed (Figure 4c,d). Clearly, the vibration excited by the mode in Figure 4d has common features with that in Figure 3. In other words, the maximum strain was mainly concentrated at the four vertexes of the ceramics, and the nodal point was in the center of the ceramics. Therefore, this mode was recognized as the shear vibration mode. Although the mode in Figure 4c had relatively large displacements at the four vertexes of the ceramics, the nodal point may be in other positions, rather than the center of the ceramics. The mode of the integral structure composed of four ceramic plates was an asymmetric Lamb wave, which was a vibration mode sensitive to the external dimension.

### 3.3. Finite Element Analysis of Four Elements with a Trapezoid Transition Layer

**Figure 5:**
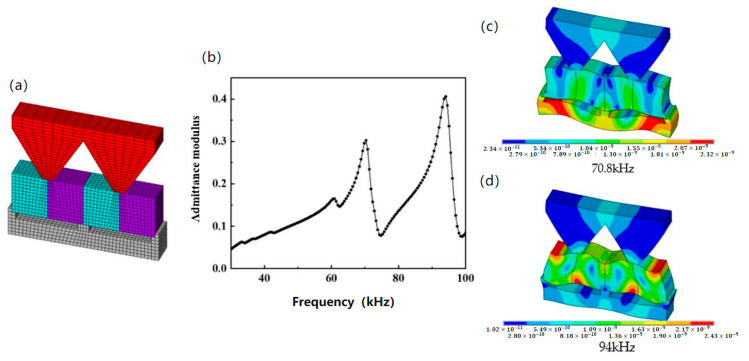
Finite element simulation results of piezoelectric oscillator. (**a**) Mesh model; (**b**) admittance modulus curve; (**c**) vibration mode@70.8kHz; (**d**) vibration mode@94kHz.

Furthermore, a third finite element model representing the bonding of four elements, a duralumin transition layer, and a positioning bar was constructed. The meshed model is shown in Figure 5a. Through exploring the trapezoid transition structure base angle, the θ of piezoelectric vibrator vibration transfer effect found that, when the θ is 120° the trapezoid transition layer on the surface vibration and minimum whole, each particle vibration almost in the same phase and the longitudinal vibration characteristic is good, which is advantageous to the acoustic radiation in the water, so the design of the base angle is 120° trapezoid transition structure. The admittance modulus curve calculation result shown in Figure 5b reflected that the piezoelectric oscillator produced two major harmonic peaks at 70.30 and 94.06 kHz in the frequency range of 30–100 kHz. The anti-resonance frequencies and electromechanical coupling factors for both harmonic peaks were 74.76 kHz, 0.37 and 99.01 kHz, 0.34, respectively. As we can see, after bonding four elements, a duralumin transition layer and positioning bar together, the electromechanical coupling factor further dropped to 0.34. The vibration mode in Figure 5d was consistent with the previous shear vibration mode, while the vibration mode in Figure 5c was consistent with the mode of asymmetric Lamb wave.

By comparing Figure 3c, Figure 4d and Figure 5d, it can be seen that the shear mode under these three conditions retained common features, namely that the maximum strain was mainly concentrated at the four vertexes of ceramics and the nodal point was in the center of ceramics. Additionally, the resonance frequency changed slightly with different boundary conditions, but the electromechanical coupling factor dropped dramatically when the conditions were different. In particular, when four elements were bonded together, the electromechanical coupling factor dropped more than 29%.

Furthermore, by observing the displacement distribution in the transition layer in Figure 5d, it can be seen that the shear vibration caused by piezoelectric ceramic was transferred into the transition layer. The vibration spread from the bottom to the upper surface of the transition layer. On the upper surface, the vibration phase and amplitude were basically consistent, thus indicating that the shear vibration mode was successfully transformed into longitudinal vibration mode through the transition layer structure. This facilitated the propagation of sound waves through water.

## 4. Preparation of Oscillator and Transducer Array

### 4.1. Preparation of Oscillator

**Figure 6:**
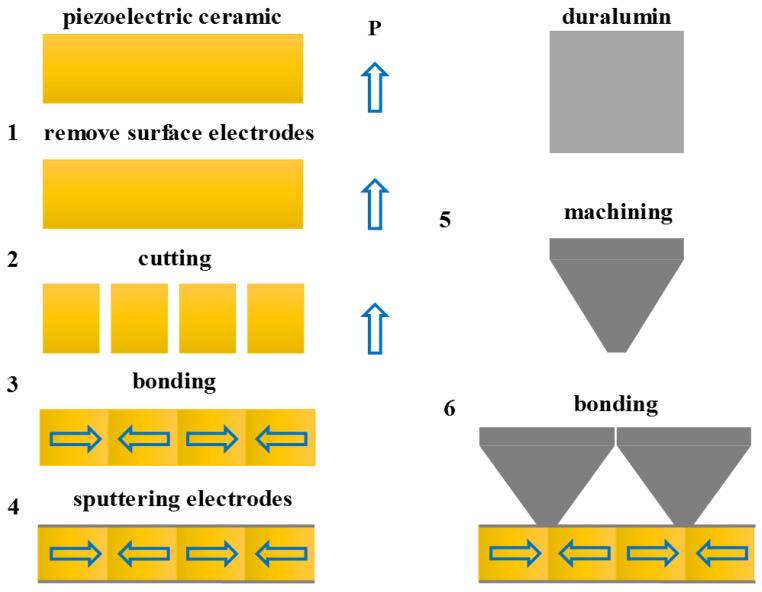
Preparation process of piezoelectric oscillator.

In this study, a piezoelectric oscillator was prepared by precise cutting and manual bonding. This process is shown in Figure 6. First, the electrodes of a PZT-5A piezoelectric ceramic block were removed; then, the ceramic block was cut into several ceramic plates of optimal sizes using a precision dicing saw (Marco Ace, Loadpoint Inc., Swindon, UK). Second, four ceramic plates with opposite polarization directions were bonded by applying resin epoxy 618 along the polarization direction. Third, silver electrode was sprayed onto the upper and lower surfaces by a high-vacuum magnetron sputtering instrument (Q300T, Quorum Technologies Ltd., Ashford, UK). Finally, the machined duralumin trapezoid transition layer was bonded to the ceramic joint by epoxy resin. In this way, the piezoelectric oscillator was prepared.

**Figure 7:**
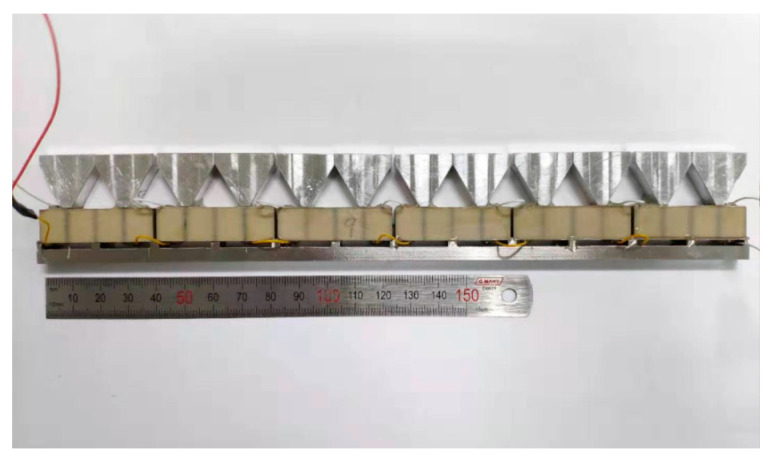
Diagrams of array elements.

Fourteen piezoelectric oscillators were made for the transducer array. Fourteen stainless steel positioning strips for fixing oscillators were fabricated to arrange oscillators reasonably at an equal distance. Six piezoelectric oscillators were bonded onto the positioning strip, one-by-one along the transversal direction. The upper and lower surfaces among different oscillators were connected by leads, and electrodes were extended from two ends, thus forming an array element (Figure 7). A total of 14 array elements were prepared. To verify the consistency of array elements, the resonance frequencies and quasi-static capacitances of these elements were measured by an impedance analyzer. Results are shown in Table 3. According to the data in Table 3, the maximum relative deviation of resonance frequencies among different array elements was 1.22%, and the maximum relative deviation of quasi-static capacitance was 1.45%. The electrical performance parameters were relatively similar, thus indicating the high consistency of array elements.

### 4.2. Preparation of Transducer Array

**Figure 8:**
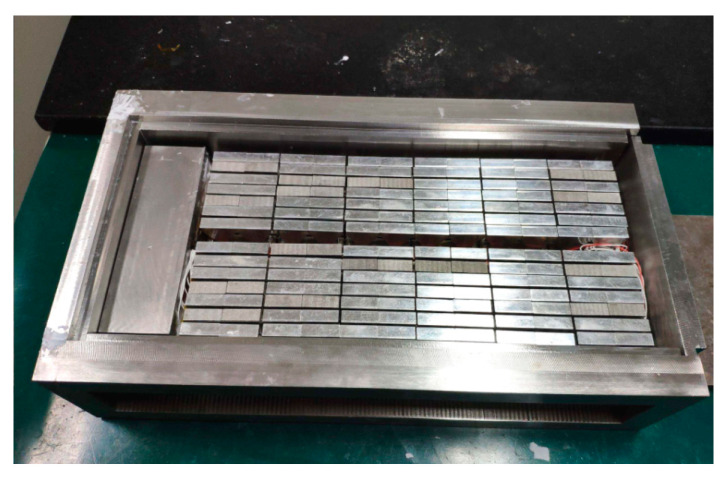
The transducer array was put in the filling mould.

**Figure 9:**
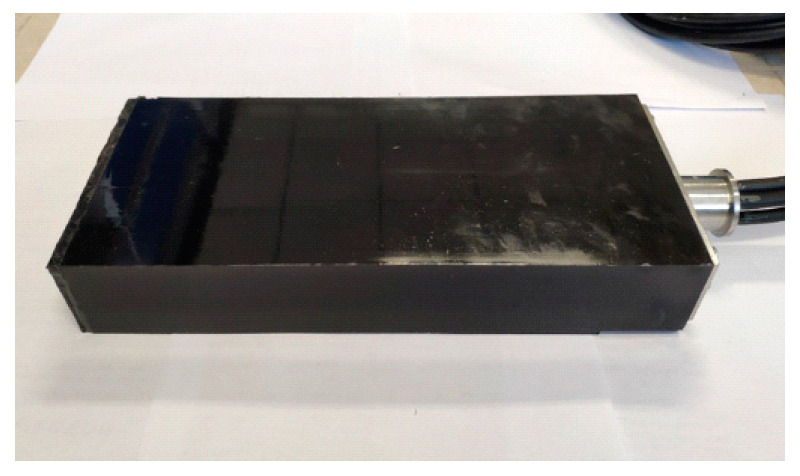
Physical pictures of the transducer array.

**Figure 10:**
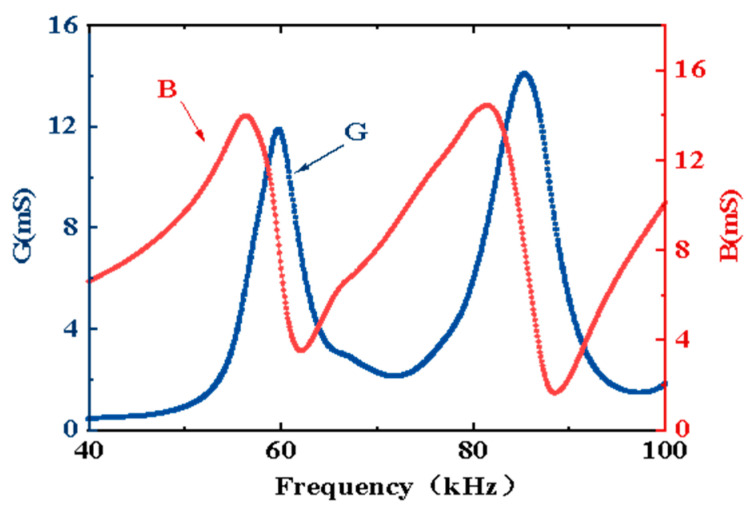
Admittance curves of the transducer array in water. B: Electric-susceptance; G: Electricconductivity.

A transducer array was made using the above array elements. The internal structure is shown in Figure 2b. First, 14 array elements were arranged reasonably at equal distances and then fixed on the positioning plate. They formed a 6 × 14 rectangular transducer array. Subsequently, all leads of the array were welded onto the output cable through an airtight box. The airtight box was equipped with a waterproof rubber gasket. Next, this array was put in a filling mold (Figure 8). Finally, the array was sealed with polyurethane (PU) water. The sealed transducer array sample is shown in Figure 9. The radiation area of this transducer array was 120 × 240 mm. The admittance curves of the transducer array in water are shown in Figure 10. The admittance curve reflected that the transducer array produced two major harmonic peaks at 59.7 and 85.3 kHz in the frequency range of 30–100 kHz.

## 5. Result

### 5.1. Test Results of Oscillator

**Figure 11:**
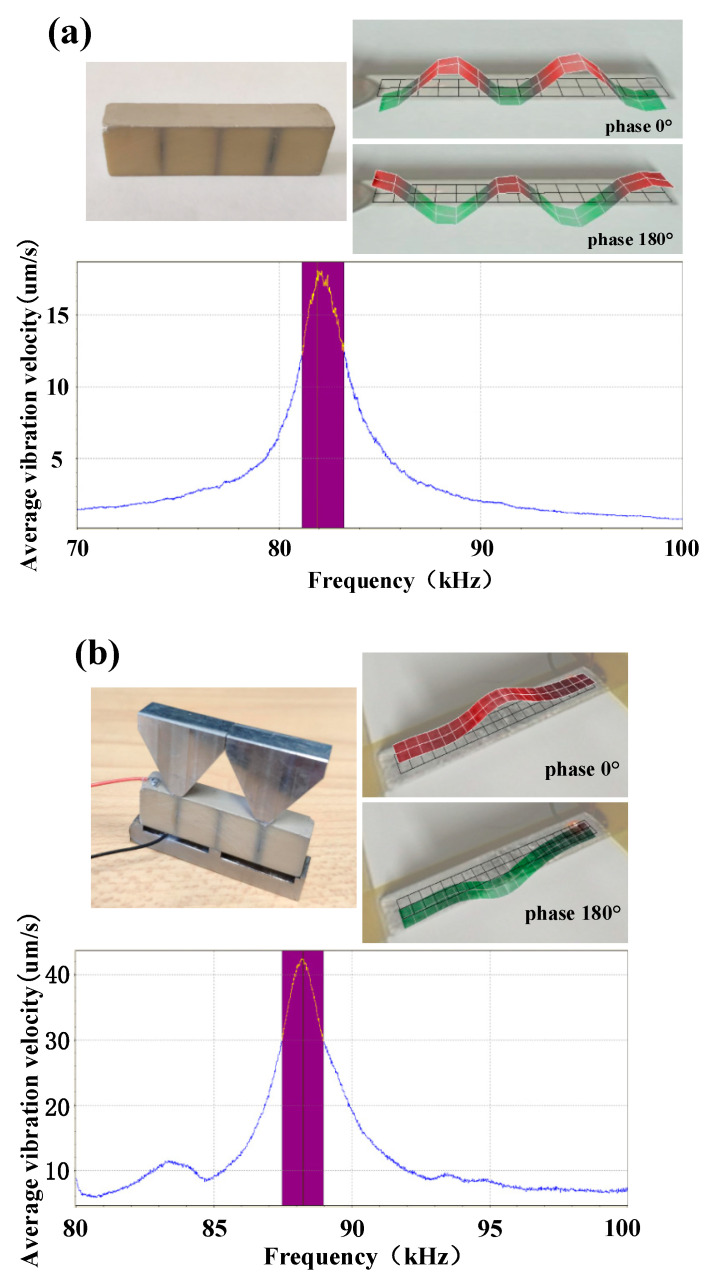
Laser vibration test results of piezoelectric oscillator. (**a**) Vibration situations on the surface of four elements and vibration velocity of the upper surface varying with frequency; (**b**) the vibration situations on the surface of four elements with the transition layer and vibration velocity of the upper surface varying with frequency.

In this study, the actual vibrations on the upper surface of four elements and four elements with the transition layer were observed by a laser Doppler vibration measuring instrument (PSV-400, Polytec Inc., Waldbronn, Germany), in order to further verify the feasibility of vibration transformation in the piezoelectric oscillator. The vibration forms on the surfaces of four bonded ceramic plates were tested; the vibration velocity of the upper surface varying with frequency was tested, and the results are shown in Figure 11a. There was one vibration velocity peak in this curve, and the frequency was 82.18 kHz. At this frequency, the vibration form was observed. It can be seen that it was a typical shear vibration form that conformed to the finite element simulation results in Figure 4d. The vibration situations on the surface of four elements with the transition layer are shown in Figure 11b. Vibrations on the surface of the transition layer were consistent with simulation results in Figure 5d. Therefore, the goal of transforming shear vibrations into vibrations on the surface of the transition layer was realized.

**Figure 12:**
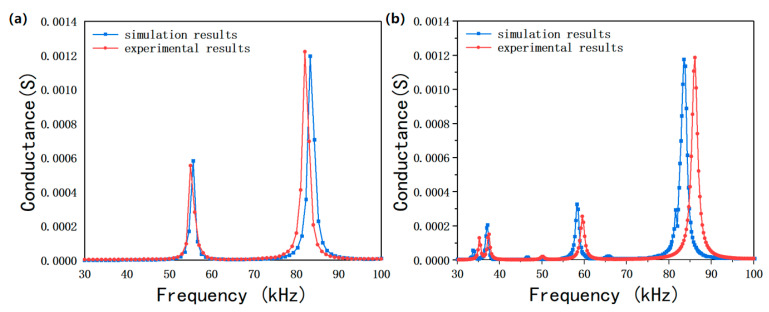
Comparison between finite element simulation and experimental results of conductance curves. (**a**) Four bonded ceramic plates; (**b**) piezoelectric oscillator.

The conductance curves of four bonded ceramic plates and a piezoelectric oscillator were measured by an HP 4294A impedance analyzer (Agilent Technologies Inc., Santa Clara, CA, USA). Anyway, these conductance curves were compared with the simulated conductance curves (Figure 12). Both conductance curves of four bonded ceramic plates and piezoelectric oscillator had harmonic peaks of the same trends as the simulated curves in the frequency range of 30–100 kHz. The simulated resonance frequencies of four bonded ceramic plates were 55.45 and 83.67 kHz, which had errors of 0.23% and 1.78%, relative to the measurement results (55.32 and 82.18 kHz). The simulated resonance frequencies of the piezoelectric oscillator were 37.20, 59.40, and 86.00 kHz, which presented errors of 0.54%, 1.94%, and 2.91%, compared to the measurement results (37, 58.25, and 83.5 kHz, respectively). It was found that the measured resonance frequencies were slightly lower than the simulated results, and the conductance corresponding to the resonance peak dropped slightly, compared to the simulated results. These results are the consequence of two factors. First, the material parameters defined in ANSYS simulation are different from practical materials to some extent. Second, the simulation model is too ideal and ignores the influence of epoxy resin adhesive, so the size parameters of each part of the device, as well as the thickness and strength of the bonding layer, may cause certain errors.

### 5.2. Test Results of the Transducer Array

**Figure 13:**
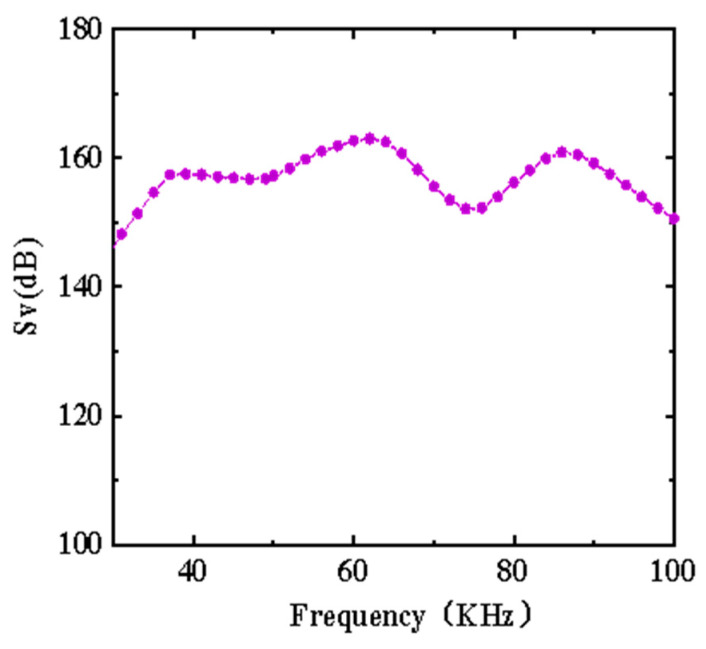
Transmitting voltage response of transducer array.

The transmitting voltage response of the above transducer array was measured using an automatic acoustic measuring system in an anechoic tank. The measurement results are shown in Figure 13. Clearly, this transducer array generated three transmitting voltage response peaks (157.5, 163, and 160.9 dB) at 39, 62, and 86 kHz, respectively. These peaks conformed to the harmonic peaks of the transducer array elements. In addition, there was a strong coupling between the transmitting voltage response peaks at 39 and 62 kHz. The transmitting voltage response curve was relatively gentle within the resonance frequency range of 37–68 kHz, and the overall fluctuation amplitude was smaller than 6 dB. If the frequency of the shear vibration mode was decreased slightly, the previous two vibration modes could be coupled into an ultra-wide band (UWB) transducer to expand the bandwidth to over 50 kHz.

The transmitting performance parameters of this transducer are compared with those of TC2084 transducer produced by Danish Reson company, as shown in Table 4. Through comparison, it can be seen that the acoustic radiation area of TC2084 transducer is 53,000 mm^2^, emission voltage response at the resonance frequency of 33 kHz reaches 166 dB, and −3 dB bandwidth is ±2 kHz. The effective radiation area of the transducer developed in this paper is 28,800 mm^2^, emission voltage response can reach 163 dB (62 kHz), −3 dB bandwidth is 12 kHz, and −6 dB bandwidth reaches 31 kHz. If the radiation area of the transducer in this paper is expanded to TC2084 transducer, the emission voltage response will be further improved.

**Figure 14:**
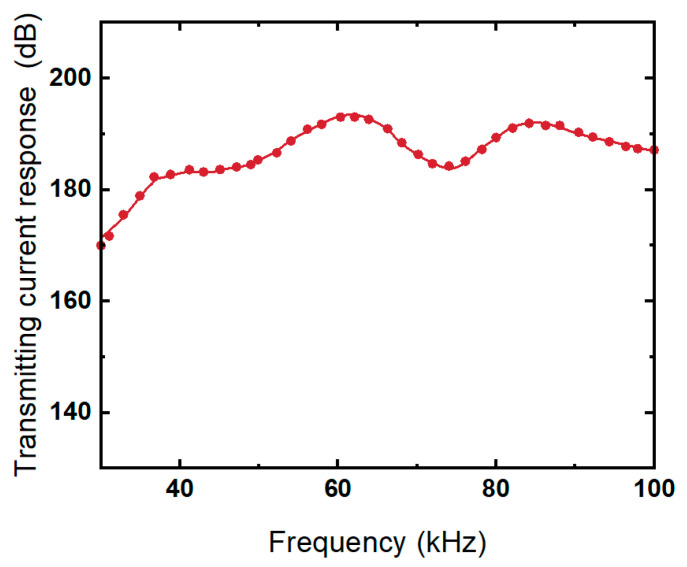
Transmitting current response of the transducer array.

The transmitting current response characterizes the intensity of the acoustic wave emitted by the transducer under a certain current. Its test principle and method are similar to the emission voltage response. Figure 14 shows the transmitting current response curve of the transducer. It can be seen that the trend of the transmitting current and transmitting voltage response curves of the transducer are basically the same, and both of them produce peaks at the same resonant frequency. At the resonance frequency of 62 kHz, the transmitting current response of the transducer reaches 193.2 dB.

**Figure 15:**
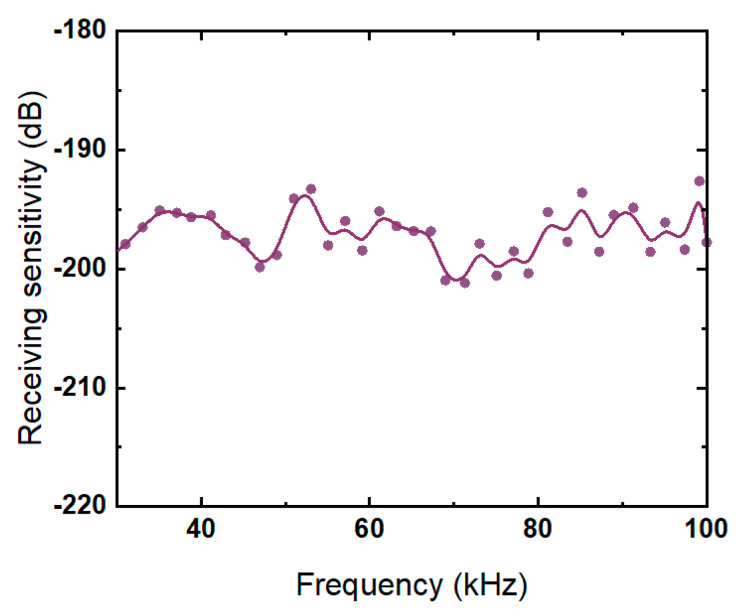
Receiving sensitivity of the transducer array.

Figure 15 shows the receiving sensitivity of the transducer. The maximum receiving sensitivity can reach −193.2 dB, and the fluctuation is about 10 dB.

## 6. Conclusions

In this study, a novel type of underwater transducer was developed using the $d_{15}$ shear vibration mode of piezoelectric ceramic and adding a trapezoid transition layer. A series of simulations on a single element, four bonding elements, and the whole structure were conducted to study how the boundary conditions affect the shear vibration. Under different conditions, the shear mode retained common features, namely that the maximum strain was mainly concentrated at the four vertexes of the ceramics and the nodal point was in the center of the ceramics. This information can help us to identify the vibration mode in the transducer. Additionally, the resonance frequency changed slightly under different conditions. The possibility of using a trapezoid transition layer to transform shear vibrations into longitudinal vibrations was verified. The shear vibrations caused by piezoelectric ceramic were effectively transferred into the transition layer, and the vibration spread from the bottom to the upper surface of the transition layer. This will make the propagation of acoustic waves into water possible. In this study, the electromechanical coupling factors of different oscillators were studied and shown to drop more than 29% when four elements were bonded together. In future studies, this kind of bonding structure will be replaced by a single element working at a high-order shear mode. According to our previous study, high-order shear vibration can have almost the same electromechanical coupling factor as the basic shear mode when the dimensions of piezoelectric ceramic are adjusted correctly [11].

The proposed transducer was prototyped and tested in water. According to experimental results, the transmitting voltage response of the transducer array reached 163 dB (62 kHz) under the radiation area of 120 × 240 mm, and the transmitting voltage response caused by $d_{15}$ shear vibration mode reached 160.9 dB at 89 kHz. The operating frequency range and bandwidth of this transducer array are 37–68 and 31 kHz, respectively. Therefore, the results demonstrate the possibility of using shear mode in an underwater transducer. This transducer design also provides a new idea for the design of wide-band, low-frequency transducers by introducing the coupling of shear mode and asymmetric Lamb waves.

## Figures and Tables

**Table 1:** Material parameters of piezoelectric oscillator.

PZT-5A						Duralumin	
$s_{11}^{E}$	1.55447 × 10^−11^	$d_{33}$	4.90 × 10^−10^	$\varepsilon_{11}^{T}/\varepsilon_{0}$	1730	Density (kg/m^3^)	2790
$s_{12}^{E}$	−4.8285 × 10^−12^	$k_{33}$	7.443 × 10^−1^	$\varepsilon_{33}^{T}/\varepsilon_{0}$	1700	Young’s modulus (GPa)	71.7
$s_{13}^{E}$	−8.0263 × 10^−12^	$d_{31}$	−2.05129 × 10^−10^	Density (kg/m^3^)	7750	Poisson’s ratio	0.35
$s_{33}^{E}$	1.61963 × 10^−11^	$k_{31}$	3.40 × 10^−1^	dielectric loss	0.02		
$s_{44}^{E}$	4.52951 × 10^−11^	$d_{15}$	5.84 × 10^−10^				
$s_{66}^{E}$	4.07464 × 10^−11^	$k_{15}$	6.85 × 10^−1^				

**Table 2:** Size parameters of piezoelectric oscillator.

Type	Bonding of Four Ceramic Plates	Type	Trapezoid Transition Layer
Length (mm)	40	Side length (mm)	Upper: 20 lower: 3
Width (mm)	12	Height (mm)	18
Thickness (mm)	7	Thickness (mm)	7

**Table 3:** Electrical performances of array elements.

Sample No.	Resonance Frequencies (kHz)	Relative Deviation (%)	Quasi-Static Capacitance (nF)	Relative Deviation (%)
1	84.61	1.22	2.342	0.17
2	83.72	0.15	2.342	0.17
3	82.82	0.93	2.342	0.17
4	84.16	0.68	2.356	0.43
5	83.17	0.51	2.32	1.11
6	83.66	0.08	2.34	0.26
7	84.16	0.68	2.365	0.81
8	83.16	0.52	2.375	1.24
9	82.67	1.11	2.33	0.68
10	83.92	0.39	2.343	0.13
11	83.17	0.51	2.352	0.26
12	84.16	0.68	2.312	1.45
13	83.13	0.56	2.361	0.64
14	83.81	0.26	2.364	0.77

**Table 4:** Comparison of emission performance of underwater acoustic transducers.

Parameters	Our Results	TC2084
Acoustic radiation area	28,800 mm^2^	53,000 mm^2^
Resonant frequency	62 kHz	33 kHz
−3 dB bandwidth	12 kHz	2 kHz
−6 dB bandwidth	31 kHz	-
Emission voltage response	163 dB	166 dB

## Data Availability

Not applicable.
